# Ground Penetrating Radar for Subsurface Utility Detection: Methods, Challenges, and Future Directions

**DOI:** 10.3390/s26092708

**Published:** 2026-04-27

**Authors:** Sijie Gao, Da Hu

**Affiliations:** Department of Civil and Environmental Engineering, Kennesaw State University, Marietta, GA 30060, USA; sgao8@students.kennesaw.edu

**Keywords:** ground-penetrating radar, utility detection, deep learning, event–utility mismatch, synthetic data, domain gap

## Abstract

**Highlights:**

**What are the main findings?**

- Event–utility mismatch limits reliable interpretation of GPR detections.
- Synthetic training data causes performance gaps in real-field conditions.
- Current models focus on hyperbola detection rather than utility inference.

**What are the implications of the main findings?**

- Shift from event detection to utility-level reasoning is needed.
- Hybrid datasets combining simulation and field data are essential.
- Physics-guided and multi-source approaches can improve reliability.

**Abstract:**

Ground-penetrating radar (GPR) has applications across many domains, including archaeology, mining, and infrastructure inspection. This review is specifically focused on urban subsurface utility mapping, where accurate detection of buried pipelines, cables, and conduits is critical for excavation safety and infrastructure management. Within this scope, two major barriers are identified: event–utility mismatch and the synthetic–field domain gap. Bibliometric analysis shows increasing reliance on deep learning, yet most methods remain limited to event-level hyperbola detection rather than utility-level inference. In real urban environments, radar responses are often affected by orientation-dependent signatures, clutter, overlapping reflections, and non-utility anomalies, making detected events difficult to map directly to physical infrastructure. In parallel, models trained on synthetic data frequently show limited field generalization because simulated radargrams do not fully reproduce soil heterogeneity, acquisition variability, and system artifacts. The review argues that future progress in urban utility mapping requires a shift toward utility-level reasoning supported by multi-sensor fusion, physics-guided learning, hybrid simulation–field datasets, and uncertainty-aware interpretation. Such advances are essential for making GPR outputs more reliable and actionable in urban engineering practice.

## 1. Introduction

The rapid expansion of urbanization has necessitated the development of complex underground infrastructure networks, including water supply systems, gas pipelines, electrical cables, and telecommunication lines [1,2]. These buried utilities constitute a lifeline of modern civil infrastructure, yet their invisible nature poses significant risks during excavation and construction activities [3,4]. Accurate detection and mapping of subsurface utilities are critical for preventing accidental damage, ensuring public safety, and minimizing economic losses associated with service interruptions [5,6]. In densely populated urban environments, the congestion of underground space exacerbates the difficulty of maintaining accurate records, often leading to reliance on outdated or incomplete as-built drawings [7,8]. Consequently, the demand for reliable non-destructive testing (NDT) methods capable of verifying subsurface conditions prior to excavation has become paramount in civil engineering practice [9,10].

Among various geophysical techniques, ground-penetrating radar (GPR) has emerged as the most widely adopted method for subsurface utility detection [11,12]. The preference for GPR stems from its ability to provide high-resolution imagery of shallow subsurface structures without disturbing the ground surface [13,14]. Unlike electromagnetic locators which are primarily effective for metallic utilities, GPR can detect both metallic and non-metallic targets, such as plastic pipes and concrete conduits, by exploiting contrasts in dielectric properties [15,16]. The technology offers rapid data acquisition and has been successfully applied in diverse scenarios ranging from urban utility mapping to archaeological prospection and road condition assessment [2,17,18,19,20]. Recent advancements in multi-channel and 3D GPR systems have further enhanced the capability to visualize subsurface features in complex environments [3,4].

Despite decades of research and technological advancement, the practical application of GPR for utility detection remains constrained by several persistent challenges [5,6]. The primary obstacle lies in the interpretation of GPR data, which is often complicated by clutter, noise, and signal attenuation caused by heterogeneous subsurface media [7,8]. In urban settings, the presence of multiple overlapping utilities and surface clutter generates complex radargrams that are difficult to decipher [9,10]. Traditionally, data interpretation has relied heavily on manual analysis by experienced operators, a process that is labor-intensive, subjective, and prone to human error [11,12]. While automated processing techniques have been developed to assist in feature extraction, their robustness often diminishes when applied to field data with varying soil conditions and noise levels [13,14].

A critical analysis of the existing literature reveals two fundamental gaps that limit the progression of GPR-based utility detection systems. First, the majority of existing detection algorithms focus on event-level detection rather than utility-level inference [15,16]. Current state-of-the-art methods, particularly those based on deep learning, are predominantly designed to identify hyperbolic reflections or discrete anomalies within a B-scan image [21,22]. While effective at locating signal signatures, these methods often fail to associate detected events with specific utility types or to reconstruct the continuous spatial geometry of the utility network [23,24]. This limitation restricts the utility of automated systems to preliminary scanning, requiring significant post-processing to generate actionable utility maps [25,26]. Second, there is an increasing reliance on synthetic data for training and validating detection models, creating a significant domain gap with field conditions [27,28]. Due to the scarcity of large-scale, labeled field datasets, many recent studies utilize numerical simulation software, such as gprMax v.3.1.7, to generate training data [29,30]. While synthetic data allows for controlled experimentation, it often fails to capture the complexity of real-world clutter, antenna coupling effects, and soil heterogeneity [31,32]. Consequently, models trained predominantly on synthetic data frequently exhibit degraded performance when deployed in actual field surveys, limiting their practical applicability [33].

This review comprehensively examines the state of the art in GPR-based underground utility detection, with a specific focus on signal processing, machine learning applications, and data acquisition strategies [34,35]. The scope encompasses an analysis of traditional migration and inversion techniques alongside recent advancements in deep learning architectures such as Convolutional Neural Networks (CNNs) and Transformers [8,36]. Furthermore, the review evaluates the efficacy of data augmentation techniques and the trade-offs between simulation-based and field-based training approaches [37,38]. The main contributions of this review are threefold: *Taxonomy of Detection Methods*: A systematic classification of existing methods based on their reasoning level and data source. *Critical Analysis of Domain Gaps*: An in-depth discussion on the limitations of synthetic data and the challenges of transferring laboratory performance to field deployment. *Future Directions*: Recommendations for developing utility-level reasoning frameworks and strategies to bridge the simulation-to-reality gap through hybrid data strategies and domain adaptation.

This review argues that progress in GPR-based utility detection is limited not by detection algorithms alone, but by data realism and the lack of utility-level reasoning. Addressing these foundational issues is essential for transitioning GPR technology from a diagnostic tool to an autonomous infrastructure mapping system.

## 2. Bibliometric Analysis of GPR-Based Utility Detection Research

### 2.1. Data Collection and Search Strategy

**Figure 1:**
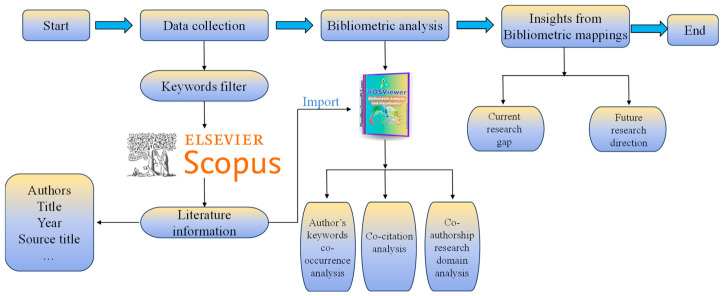
Overview of the bibliometric analysis framework.

To gain an in-depth understanding of domain knowledge pertaining to the subject of GPR, this study employs a hybrid literature review methodology that has been extensively validated and applied in prior research [39,40]. As illustrated in Figure 1, the implementation of this methodology comprises three sequentially integrated phases: First, data collection is conducted through systematic retrieval of relevant publications from selected academic databases. Second, bibliometric analysis is performed to construct a scientific knowledge map of the extant literature, thereby identifying research hotspots and evolutionary trends. Third, in-depth qualitative synthesis of key subtopics is carried out to comprehensively evaluate the current research landscape and formulate forward-looking recommendations for future research directions. This methodological framework ensures systematicity and rigor throughout the research process, providing a reliable structure for the organized exploration of domain knowledge.

Data quality constitutes the cornerstone of literature review reliability. Consequently, prior to conducting bibliometric analysis and qualitative synthesis, this study established a comprehensive database and implemented a stringent search protocol. Compared with IEEE Xplore and Web of Science, Scopus was the most suitable database for this review because the topic of GPR-based underground utility detection sits at the intersection of civil engineering, geophysics, remote sensing, non-destructive testing, and data-driven methods. IEEE Xplore is highly valuable for technical and conference-heavy literature in electrical engineering, computing, and related technologies, while Web of Science is especially strong in rigorous citation indexing across a carefully curated multidisciplinary core collection [41,42]. By contrast, Scopus offers broad interdisciplinary coverage. Its extensive coverage of construction engineering research, broad inclusion of interdisciplinary publications and its significant representation of high-impact peer-reviewed journals, making it sufficient as a single database for building a balanced and coherent review corpus. The search strategy rigorously identified core keywords aligned with the GPR theme. Publications indexed in Scopus between 2016 and 2025 (published) were retrieved, a temporal scope deliberately chosen to encompass both the contemporary relevance and scholarly representativeness of GPR research evolution. The specific search string is formulated as follows:

TITLE-ABS-KEY ((“ground penetrating radar” OR “ground-penetrating radar” or “GPR”) AND (“imag*” OR “Hyperbo*” OR “B scan*” or “Bscan*” OR “radargram*” OR “profile*” or “scan*”) AND (“utilit*” or “pipe*” or “cable*” or “conduit*” or “infrastructure” or “buried”)) AND (“detect*” OR “map*” OR “locat*” OR “recogni*” OR “identif*” OR “extract*” OR “interpret*” OR “characteri*” OR “estimat*” OR “segment*” OR “reconstruct*”) AND PUBYEAR > 2015 AND PUBYEAR < 2026 AND (LIMIT-TO (DOCTYPE, ”ar”) OR LIMIT-TO (DOCTYPE, ”cp”)) AND (LIMIT-TO (LANGUAGE, ”English”)).

The retrieval strategy was meticulously designed to comprehensively capture relevant literature on GPR-based underground utility detection while maintaining specificity and minimizing noise from unrelated applications. The query structure systematically addresses four critical dimensions: First, the core technology is represented by multiple variants of “ground penetrating radar” and its acronym “GPR” to ensure complete coverage across terminology preferences in the literature. Second, the data representation layer incorporates comprehensive terms for GPR output formats including “imag*”, “Hyperbo*” (encompassing hyperbolic signatures and hyperbolas), “B scan*”, “Bscan*”, “radargram*”, “profile*”, and “scan*” to capture diverse methodological approaches regardless of their specific data processing terminology. Third, the target object dimension employs a broad yet focused set of terms (“utilit*”, “pipe*”, “cable*”, “conduit*”, “infrastructure”, “buried”) that collectively encompass the full spectrum of underground utilities while excluding unrelated GPR applications such as archaeological investigations or concrete inspection. Fourth, the task dimension incorporates an extensive array of action verbs (“detect*”, “map*”, “locat*”, “recogni*”, “identif*”, “extract*”, “interpret*”, “characteri*”, “estimat*”, “segment*”, “reconstruct*”) to capture the complete methodological landscape from basic detection to advanced reconstruction tasks. The temporal constraint (2016–2025) ensures relevance to contemporary research while capturing the critical period of algorithmic advancement in this field. By combining these dimensions through precise Boolean logic and limiting results to peer-reviewed articles and conference proceedings in English, this retrieval strategy achieves optimal balance between comprehensiveness and specificity, effectively isolating literature that directly addresses the core research questions while filtering out peripheral applications that would dilute analytical focus. The systematic application of inclusion/exclusion criteria, coupled with the demographic representativeness of the resultant corpus, establishes a robust foundation for subsequent analytical phases.

### 2.2. Publication Trends and Research Growth

**Figure 2:**
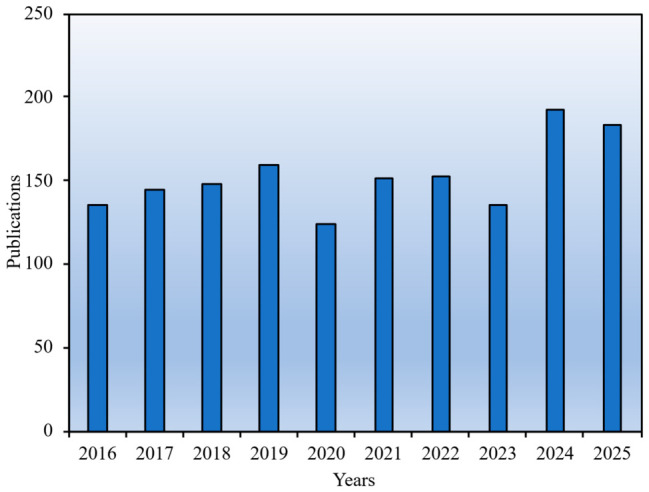
Number of annual publications from 2016 to 2025.

Adhering to the aforementioned data collection protocol, this study retrieved a total of 1525 eligible publications from the Scopus database within the defined temporal scope (2016–2025), comprising 851 peer-reviewed journal articles and 674 peer-reviewed conference papers. Figure 2 illustrates the temporal evolution of annual GPR-related publications from 2016 to 2025. The publication output remained relatively stable during the initial period (2016–2018), with annual counts ranging from 135 to 150. A phase of moderate fluctuation appeared from 2019 to 2022, with output increasing to approximately 160 publications in 2019, declining to 125 in 2020, and then recovering to around 155 in both 2021 and 2022. After a slight decrease to 135 publications in 2023, the annual output rose markedly to 195 in 2024 and remained high at 185 in 2025. Overall, these data suggest a broadly sustained research presence with a recent increase in publication output. However, short-term variations, especially those observed around 2020–2022, should be interpreted cautiously, as they may also reflect external disturbances such as the COVID-19 pandemic, which affected research activities, field investigations, international collaboration, and publication workflows across many disciplines. Therefore, annual publication counts can indicate the general development of the field, but they should not be treated as a standalone measure of research activity without considering broader contextual factors.

**Figure 3:**
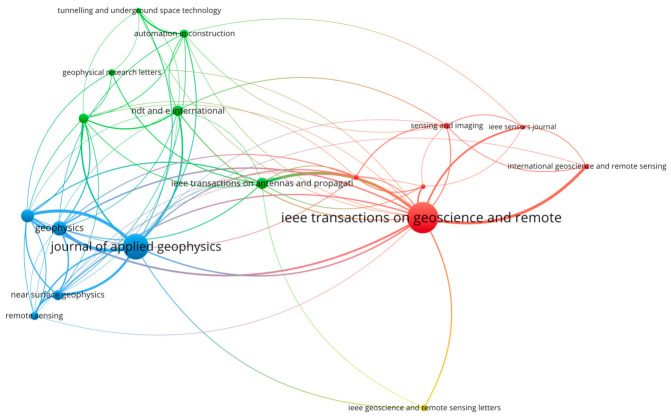
Co-citation network of publication sources.

To construct the co-citation network of publication sources, a minimum citation threshold was applied per source within VOSviewer (version 1.6.20) [43,44]. As visualized in Figure 3, node dimensions scale proportionally with citation frequency, with IEEE Transactions on Geoscience and Remote Sensing emerging as the network’s dominant node. VOSviewer’s clustering algorithm automatically groups nodes of identical color into thematic clusters, revealing three distinct communities: Cluster 1 (red; right quadrant): Dominated by IEEE Transactions on Geoscience and Remote Sensing, this community concentrates on remote sensing methodologies, sensor instrumentation, and signal processing (IEEE Sensors Journal); Cluster 2 (green; upper-left quadrant): Centered on Automation in Construction and NDT & E International, it emphasizes civil engineering applications, non-destructive testing, and underground space technology; and Cluster 3 (blue; lower-left quadrant): Anchored by Journal of Applied Geophysics and Geophysics, it focuses on applied geophysical methods, near-surface investigation, and theoretical frameworks.

This topological configuration delineates disciplinary boundaries and cross-cutting knowledge linkages within GPR research. The spatial segregation of clusters (coupled with their thematic coherence) provides an empirical foundation for subsequent bibliometric interpretation.

**Figure 4:**
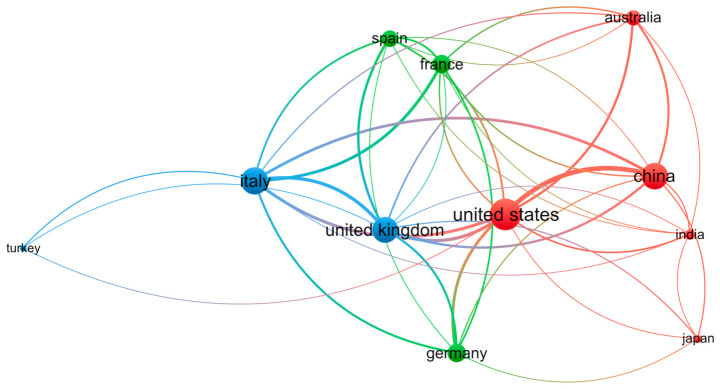
Co-authorship network in the GPR research countries.

**Figure 5:**
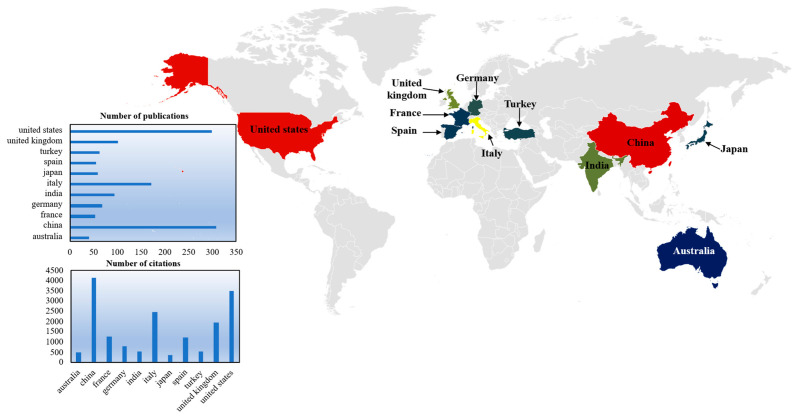
Research countries in the field of GPR and the number of publications and citations.

This study conducted a co-authorship analysis by geographic region (country/territory) to map the global collaboration network structure. Using VOSviewer’s co-authorship module, bibliometric data were filtered with dual thresholds: minimum document count (≥40 publications) and minimum total citations (≥20 citations) per region. Of the initial pool of countries, 11 satisfied these criteria. The resulting collaboration network is visualized in Figure 4, with comprehensive bibliometric indicators detailed in Figure 5. As visualized in Figure 4, node dimensions scale proportionally with publication volume, with China and the United States emerging as the network’s dominant nodes. The node color gradient reflects the average publication year (2016–2024), indicating the temporal evolution of research activity. VOSviewer’s layout algorithm spatially organizes nodes based on link strength, revealing two primary collaborative clusters: Cluster 1 (Right quadrant): Centered on the United States and China, this group includes Australia, India, and Japan, indicating strong trans-Pacific and Asian research synergies; and Cluster 2 (Left/Center quadrant): Anchored by Italy and the United Kingdom, this cluster comprises France, Spain, Germany, and Turkey, reflecting a cohesive European research bloc.

As Figure 5 demonstrated, quantitative analysis reveals distinct productivity-impact profiles: China leads in volume (307 documents) and influence (4146 total citations); the United States follows closely with 298 documents and 3483 citations; and Italy demonstrates significant output (172 documents, 2463 citations). Notably, European nations such as France (54 documents) and Spain (56 documents) exhibit high citation impact (1277 and 1198 citations respectively) relative to their publication volume.

This topological configuration delineates the global distribution of GPR scholarship. The spatial proximity of major contributors (United States, Italy, China) provides empirical evidence of a highly integrated global research network where knowledge exchange transcends geographic boundaries.

### 2.3. Keyword Co-Occurrence and Research Clusters

**Figure 6:**
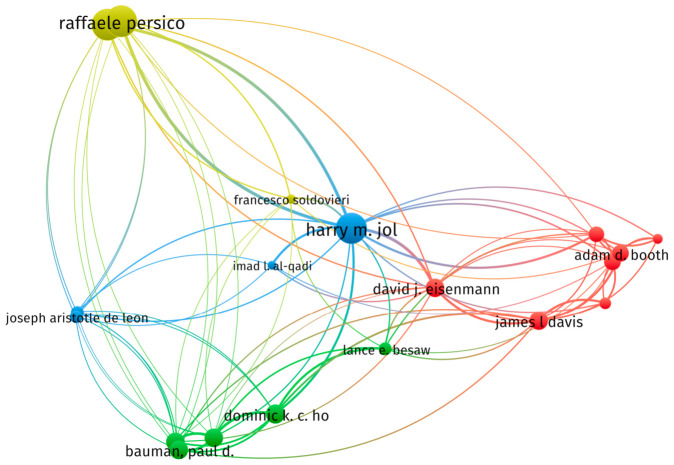
Co-citation network of authors.

To delineate the intellectual structure of the field, an author co-citation analysis was performed using VOSviewer (Figure 6). Employing fractional counting to mitigate multi-author bias, a minimum citation threshold of 10 was imposed, yielding a network of pivotal contributors. As depicted in Figure 6, node magnitude correlates with citation frequency, identifying Harry M. Jol and Raffaele Persico as the most influential anchors. The clustering algorithm partitions the network into four thematic communities, where intra-cluster connectivity reflects shared research paradigms: Cluster 1 (Yellow; upper-left): Anchored by Raffaele Persico [45] and Francesco Soldovieri [46], this group emphasizes theoretical electromagnetics, inverse scattering problems, and advanced data processing frameworks; Cluster 2 (Blue; central): Dominated by Harry M. Jol [47] and Imad L. Al-Qadi [48], this community focuses on civil infrastructure applications, particularly pavement assessment and subsurface utility mapping; Cluster 3 (Red; right): Centered on David J. Eisenmann [49], James L. Davis [50], and Adam D. Booth [51], it represents methodological advancements in object detection, signal processing; and Cluster 4 (Green; lower): Led by Dominic K. C. Ho [52], Paul D. Bauman [53], and Lance E. Besaw [54], this cluster specializes in NDT, concrete inspection, and near-surface geophysical characterization.

This topological configuration transcends mere author mapping—it empirically delineates the field’s intellectual architecture. High-co-citation nodes (Harry M. Jol and Persico) function as knowledge anchors whose work bridges theoretical and applied domains. The spatial segregation of thematic communities, validated through citation-weighted connectivity, provides a structural framework for tracing epistemic evolution pathways in ground-penetrating radar research.

**Figure 7:**
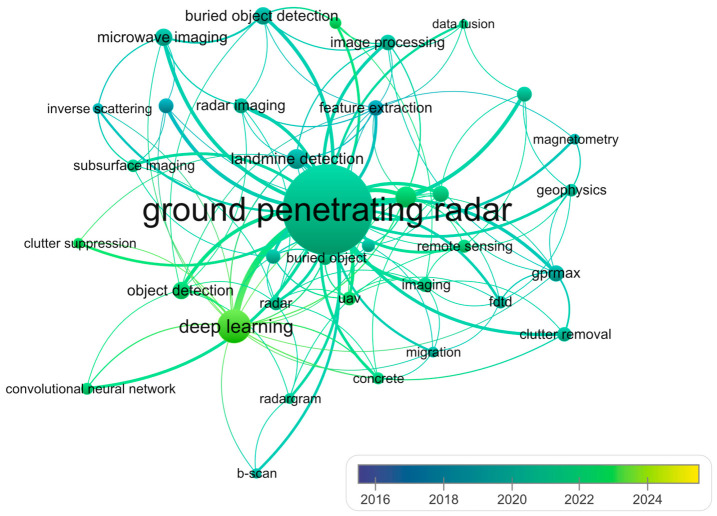
Timeline-overlayed network of author keywords co-occurrence.

To elucidate the thematic evolution and emerging frontiers in GPR research, this study constructs a keyword co-occurrence network using VOSviewer (Figure 7), where node dimensions correspond to term frequency and color gradients encode the average publication year (2016–2025). The results indicate that foundational terminology, specifically “ground penetrating radar”, serves as the network’s central hub (with gpr, ground penetrating radar, and ground-penetrating radar consolidated under “ground penetrating radar” in the figure), reflecting sustained scholarly attention throughout the study period. Traditional methodological keywords, such as “electrical resistivity tomography”, “signal processing”, and “landmine detection”, exhibit cooler color tones (blue/teal), corresponding to earlier average publication years (2016–2019) and establishing the field’s historical baseline.

In contrast, keywords associated with intelligent automation and advanced computing are concentrated in warmer hues (green/yellow), highlighting their recent proliferation. Notably, “deep learning” and “machine learning” emerge as dominant recent themes, alongside specific architectural terms like “convolutional neural network” and platform innovations such as “uav”. Furthermore, application-specific terms like “object detection” and “buried object detection” demonstrate strong linkages to these AI-driven methods. Overall, this visualization not only systematically maps the temporal distribution of research focus but also quantitatively captures the paradigm shift in GPR scholarship from conventional geophysical signal processing toward data-driven, intelligent detection frameworks, thereby providing empirical evidence for the discipline’s current trajectory.

### 2.4. Insights from Bibliometric Mappings

The bibliometric evidence accumulated in Section 2.1, Section 2.2 and Section 2.3 permits a broader interpretation of the field’s trajectory, revealing three critical dynamics shaping the future of GPR-based utility detection. By synthesizing publication trends, collaboration networks, and keyword evolution, distinct patterns emerge regarding methodological preferences, data generation strategies, and the alignment between academic output and field requirements.

The keyword co-occurrence network (Figure 7) provides compelling evidence of a paradigmatic transition from traditional signal processing techniques to data-driven methodologies. The prominence of “deep learning” and “machine learning” as recent research themes contrast sharply with the earlier emphasis on “signal processing” and “migration”. This shift is temporally corroborated by the color gradient in Figure 7, where AI-related terminology clusters in the 2022–2024 period, while conventional processing methods exhibit earlier average publication years (2016–2019). The author co-citation network further substantiates this transition. While clusters centered on authors such as Persico and Soldovieri maintain an emphasis on theoretical frameworks and electromagnetic modeling, the emerging linkages to application-specific terms like “object detection” and “buried object detection” demonstrate that contemporary research increasingly prioritizes automated interpretation over manual signal analysis. This methodological evolution reflects broader trends in computational intelligence, wherein the availability of large-scale datasets and advances in GPU computing have rendered data-hungry algorithms increasingly viable for GPR applications. However, the relatively modest occurrence counts for specific deep learning architectures, such as “convolutional neural network”, suggest that the field remains in an exploratory phase, with researchers experimenting with diverse algorithmic approaches rather than converging on established best practices.

Concurrently, the bibliometric data reveal growing attention to computational modeling and synthetic data generation. The presence of “gprmax” and “fdtd” (finite-difference time-domain) in the keyword network indicates sustained interest in electromagnetic simulation tools for generating synthetic radargrams. These simulation-based approaches serve dual purposes: validating theoretical models and augmenting limited field datasets for algorithm training. The co-citation network of publication sources (Figure 3) provides additional context for this trend. Clusters dominated by IEEE Transactions on Geoscience and Remote Sensing and Proceedings of SPIE emphasize theoretical frameworks and antenna propagation—research areas that frequently employ numerical modeling and synthetic data generation. The strong citation performance of foundational texts on inverse scattering and data processing further underscores the field’s continued engagement with forward modeling. However, the relatively low occurrence of explicit terms related to “data augmentation” in the keyword network suggests that simulation-based approaches remain primarily confined to methodological validation rather than serving as primary data sources for algorithm development. This observation aligns with the persistent emphasis on field-derived data evident in keywords such as “concrete” and “geophysics”, which imply empirical investigation rather than purely computational studies.

A critical insight emerging from the bibliometric analysis is the apparent disconnect between prevalent research themes and the practical requirements of utility detection in operational contexts. First, the keyword distribution reveals disproportionate attention to certain application domains that may not align with the most pressing industry needs. “Landmine detection” exhibits higher frequency than utility-specific terms, reflecting the field’s historical roots in defense and humanitarian demining applications rather than civil infrastructure management. While methodological advances in landmine detection may transfer to utility mapping, the distinct characteristics of these domains (including target depth, material composition, and environmental conditions) limit direct applicability. Second, the temporal analysis indicates that emerging research frontiers emphasize algorithmic sophistication over practical deployment considerations. Keywords such as “deep learning” and “feature extraction” dominate recent literature, yet terms related to field implementation challenges (including “real-time processing,” “portable systems,” or “on-site interpretation”) remain conspicuously absent from the high-frequency keyword set. This imbalance suggests that academic research prioritizes methodological innovation and benchmark performance over operational feasibility and user accessibility. Third, the author co-citation network reveals thematic segregation between theoretical/algorithmic research and applied geophysical investigation. While such specialization is intellectually productive, the limited cross-cluster connectivity may impede the translation of algorithmic advances into field-deployable solutions. Practitioners requiring robust, interpretable tools for routine utility mapping may find limited guidance in the literature emphasizing novel neural architecture or theoretical electromagnetic modeling. Collectively, these observations suggest that while GPR research has achieved substantial methodological sophistication, particularly in data-driven approaches and computational modeling, the field would benefit from increased attention to implementation challenges, interpretability requirements, and the heterogeneous conditions characteristic of real-world utility detection scenarios.

## 3. Hyperbolic Responses in GPR B-Scans for Utility Detection

### 3.1. Formation of Hyperbolic Signatures in B-Scans

The formation of hyperbolic signatures originates from the geometry of electromagnetic wave propagation as the GPR antenna moves along a survey line above a point-like or cylindrical subsurface object. When the antenna transmits a short pulse of electromagnetic energy into the ground, the wavefront propagates spherically through the subsurface medium. Upon encountering a dielectric contrast, such as the interface between soil and a buried pipe, the energy partially reflects back toward the receiving antenna [55].

**Figure 8:**
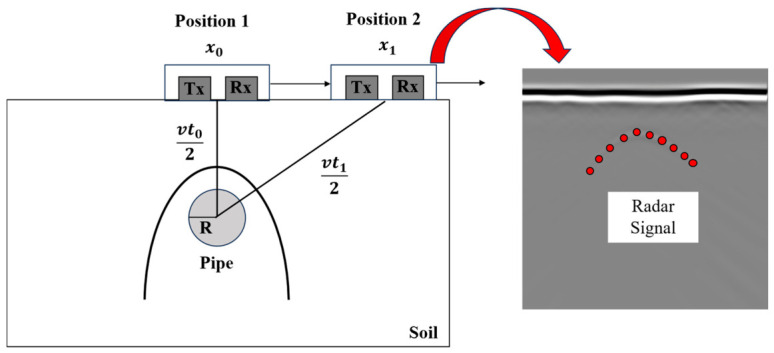
Geometry of the soil structure including a buried pipe.

As the antenna position changes relative to the target (Figure 8), the two-way travel time of the reflected signal varies according to the geometric relationship between source, target, and receiver. When the antenna is directly above the object, the travel path is shortest (vertical incidence), producing the minimum two-way travel time observed at the apex of the hyperbolic radar signal. As the antenna moves laterally away from the target’s projection on the surface, the travel path lengthens following the Pythagorean relationship, resulting in progressively longer two-way travel times that trace the characteristic downward-opening hyperbolic curve in the time–distance domain of the B-scan image [21,56].

Mathematically, for a target below the surface, the GPR data are often formulated as Equation (1):

(1) $$\frac{{\left(t_{0}+\frac{2R}{v}\right)}^{2}}{{\left(t_{1}+\frac{2R}{v}\right)}^{2}}-\frac{{\left(x_{0}-x_{1}\right)}^{2}}{{\left(\frac{vt_{0}}{2}+R\right)}^{2}}=1$$

where *t*_1_ represents the two-way travel time, *R* is the radius of the pipe, *x*_1_ is the horizontal position, and the velocity of propagation is denoted by *v*, *x*_0_ and *t*_0_ represent the position and two-way travel time when the GPR is above the pipe [5].

This equation explicitly demonstrates the hyperbolic nature of the reflection pattern. In practice, cylindrical utilities such as pipes generate similar hyperbolic signatures when the survey line crosses perpendicular to the pipe orientation; however, deviations from perpendicular transects produce distorted or partial hyperbolic patterns that complicate interpretation [57,58]. The clarity and completeness of hyperbolic signatures are further influenced by subsurface conditions including soil heterogeneity, moisture content, electrical conductivity, and the presence of clutter from adjacent utilities or natural subsurface features [27].

### 3.2. Why Hyperbolic Responses Became the Dominant Utility Proxy

Hyperbolic signatures have emerged as the dominant proxy for utility detection in GPR interpretation for several compelling reasons that span perceptual, algorithmic, and practical domains.

First, the hyperbolic pattern presents a geometrically distinctive and consistent feature that stands out against the typically horizontal layering of natural soil stratigraphy in radargrams. This clear geometric pattern provides an intuitive visual cue that experienced interpreters can rapidly identify even in moderately noisy data [3,37]. Field practitioners have historically relied on this visual recognition capability, establishing hyperbola identification as a cornerstone of GPR interpretation practice for utility detection [30]. The interpretability of hyperbolas has been further enhanced through migration processing techniques that collapse the hyperbolic diffractions to their point of origin, thereby improving spatial resolution and depth accuracy [22,59].

Second, the mathematical regularity of hyperbolic signatures makes them particularly amenable to algorithmic detection and parameter extraction. Early automated approaches employed Hough transforms and curve-fitting algorithms to identify hyperbolic patterns and extract geometric parameters such as depth and radius [36,60]. More recently, machine learning techniques have significantly advanced hyperbola detection capabilities. CNNs have demonstrated remarkable success in classifying hyperbolic patterns with accuracies exceeding 94% on both synthetic and field data [61,62]. The development of specialized architectures, including YOLO (You Only Look Once) variants with attention mechanisms [17], three-dimensional CNNs for volumetric data analysis [32], and integrated contouring approaches [25], has further improved detection reliability while reducing false alarm rates in complex urban environments with multiple overlapping utilities [63].

Third, hyperbolic signatures provide a direct physical link to quantifiable subsurface parameters. Through velocity analysis and hyperbola fitting procedures, the apex position yields target depth, while the curvature relates to target size and electromagnetic properties of both the object and surrounding medium [26,64]. However, the accuracy of these estimates depends strongly on the underlying travel-path model. Xie et al. showed that treating a common-offset GPR antenna as a point source can introduce bias in velocity estimation, and that incorporating antenna separation and target radius into a nonlinear fitting framework improves positioning reliability [65]. Advanced inversion techniques such as full-waveform inversion (FWI) can further refine estimates of pipe diameter and even infer infilling materials (air, water, sediment) by matching observed hyperbolic characteristics with forward-modeled responses [33,66].

Despite these advantages, hyperbolic interpretation faces inherent limitations. Non-perpendicular survey orientations produce incomplete or distorted hyperbolas that may be misinterpreted or missed entirely [57]. Small-diameter utilities relative to the radar wavelength may generate weak or ambiguous signatures [34]. Additionally, complex subsurface conditions, including heterogeneous soils, closely spaced utilities, and scattering from rocks or debris, can obscure hyperbolic patterns or create false hyperbolic-like features [67,68]. Nevertheless, the combination of physical interpretability, algorithmic tractability, and established field practice has cemented hyperbolic signatures as the predominant feature for utility detection in GPR applications, with ongoing research focused on enhancing robustness through multimodal sensing integration and advanced machine learning approaches [69,70].

## 4. Computational Approaches for GPR Event Detection

Computational methods for GPR event detection have evolved substantially over the past two decades, yet they remain intermediate analytical tools rather than definitive solutions to subsurface interpretation challenges. These approaches facilitate the identification of reflection patterns, particularly hyperbolic signatures associated with buried utilities, but cannot resolve fundamental ambiguities arising from complex subsurface conditions such as soil heterogeneity, electromagnetic clutter, velocity variations, and geometric distortions [64,71]. The distinction between “detecting” a reflection event and “interpreting” its physical origin remains critical: improved detection algorithms enhance signal visibility but do not eliminate the inherent non-uniqueness of electromagnetic inverse problems in realistic field environments [72,73]. Beyond feature extraction and pattern recognition, some studies have framed GPR subsurface sensing as a fully nonlinear inverse-scattering problem. In this line of work, multifrequency and multiresolution inversion strategies have been proposed to improve image fidelity, especially when linearized approximations become inadequate for complex buried targets [74].

### 4.1. Classical Signal and Image Processing Methods

Early computational strategies for GPR event detection relied primarily on deterministic signal and image processing techniques. Thresholding operations applied to amplitude envelopes or energy maps enabled basic separation of reflections from background noise, though performance degraded significantly in low signal-to-noise ratio (SNR) conditions common in urban subsurface surveys [68]. Frequency-domain filtering, including bandpass filtering to suppress antenna ringing and migration filters to collapse diffractions, provided modest improvements in hyperbola clarity but often introduced artifacts when velocity models deviated from actual subsurface conditions [22,59].

**Figure 9:**
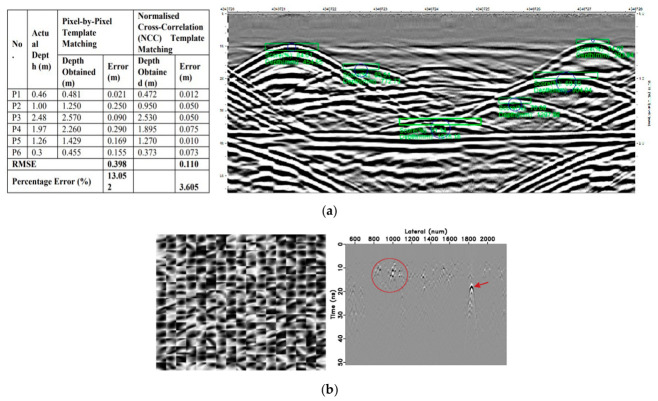
(**a**) The results of depth obtained by using pixel-by-pixel (direct comparison) template matching and pipe detection using automatic detection system for GPR dataset [36]; (**b**) learned dictionary image of the field GPR data with a size of 11 × 11 samples and diffraction extraction profile [76].

Template matching and curve-fitting approaches represented a more geometrically informed strategy (Figure 9). Hyperbolic templates parameterized by depth, velocity, and target size were cross-correlated with B-scan data to identify candidate reflections [36]. Simultaneous perturbation algorithms further refined geometric parameter extraction by fitting hyperbolic models to detected events [60]. While mathematically elegant, these methods exhibited strong sensitivity to survey geometry, for example, non-perpendicular transects across pipes produced distorted or truncated hyperbolas that violated template assumptions, leading to missed detections or false positives [56,75]. Dictionary learning techniques attempted to address this limitation by adaptively extracting diffraction patterns from data without strict geometric priors, yet they remained constrained by the requirement for representative training dictionaries and struggled with overlapping reflections from congested utility corridors [76].

### 4.2. Machine Learning with Handcrafted Features

The introduction of machine learning classifiers marked a shift toward data-driven event detection, though reliance on handcrafted features preserved significant site dependence. Feature engineering typically involved extracting statistical descriptors (kurtosis, entropy), textural measures (gray-level co-occurrence matrices), or transform-domain coefficients (wavelet packet energies) from localized image patches [3,77]. Support vector machines and shallow neural networks then classified patches as “hyperbola” or “background” based on these features [78].

Despite achieving moderate success on controlled datasets, these approaches revealed critical limitations in transferability. Features optimized for sandy soils with low conductivity often failed in clay-rich or moisture-saturated environments where electromagnetic attenuation altered reflection morphology [79]. Similarly, classifiers trained on metallic pipes exhibited degraded performance on non-metallic utilities due to differences in reflection strength and waveform characteristics [37]. Multi-objective genetic algorithms attempted to optimize feature subsets for robustness across sites, yet fundamental trade-offs persisted: features sensitive to subtle hyperbolic curvature also responded strongly to clutter from rocks or roots, while clutter-resistant features often missed small-diameter utilities [80]. This site-specific calibration burden undermined practical deployment, as each new survey location effectively required retraining, a prohibitive requirement for routine utility mapping operations [12].

### 4.3. Deep Learning for Event-Level Detection

Deep learning architectures have substantially advanced event-level detection capabilities through hierarchical feature learning that bypasses manual feature engineering. CNNs trained on synthetic or field-collected GPR data demonstrated remarkable proficiency in hyperbola identification, with reported detection accuracies exceeding 90% on benchmark datasets [61,62]. Architectural innovations further enhanced performance: attention mechanisms improved focus on reflection apex regions while suppressing clutter [81]; U-Net variants enabled pixel-wise segmentation of hyperbolic signatures [82]; and transformer-based models captured long-range contextual dependencies critical for distinguishing overlapping reflections [83].

Object detection frameworks adapted from computer vision, particularly YOLO variants and Multi-view CNN, extended capabilities to simultaneous localization and classification of multiple subsurface targets [17,84]. Cycle-consistent generative adversarial networks (CycleGANs) showed promise in clutter suppression by learning mappings between cluttered and clean GPR domains without paired training data [85]. End-to-end keypoint detection networks further refined geometric parameter estimation by directly predicting hyperbola apex coordinates and curvature parameters [86]. A comparison in the performance and techniques employed by the different studies have been outlined in Table 1.

Nevertheless, the disparity between laboratory-reported accuracy and field deployment performance remains pronounced. Three primary factors explain this gap:

Domain shift between synthetic training data and field conditions: Most deep learning models rely heavily on synthetic data generated via gprMax or similar electromagnetic simulators to overcome limited annotated field datasets [87,88]. While synthetics capture idealized hyperbolic responses, they often omit realistic complexities, such as soil layering irregularities, root networks, or construction debris, that dominate field radargrams [89]. Domain adaptation techniques partially mitigate this issue but cannot fully compensate for unmodeled physical phenomena [90].

Contextual ambiguity unresolved by pixel-level analysis: Deep networks excel at recognizing hyperbolic shapes but cannot distinguish whether a reflection originates from a utility pipe, a rock, a void, or a soil interface without additional contextual information [91]. Field validation studies revealed that up to 40% of algorithmically detected “pipes” corresponded to non-utility objects when ground-truth excavation was performed [92].

Sensitivity to acquisition geometry and preprocessing choices: Detection performance degrades substantially when survey parameters (antenna height variations, non-linear transects) or preprocessing pipelines (background removal methods, gain functions) differ from training conditions [93,94]. Few-shot learning approaches aim to address this through rapid adaptation but require representative exemplars from the target site, reintroducing the site-dependence problem these methods sought to eliminate [95].

Critically, even perfect event detection cannot resolve the fundamental ambiguity inherent in GPR interpretation as a detected hyperbola provides geometric constraints on target depth and size but reveals little about material composition, structural integrity, or functional status without complementary information [96]. Velocity uncertainties of ±10%, common in heterogeneous urban soils, translate to depth estimation errors exceeding 15 cm at 1 m depth, potentially mispositioning utilities relative to excavation zones [21]. Similarly, overlapping hyperbolas from parallel utilities separated by less than a wavelength cannot be resolved regardless of detection algorithm sophistication due to the diffraction limit of the radar system itself [11].

## 5. Event–Utility Mismatch in Real-World GPR Data

The fundamental challenge in GPR utility detection lies not in identifying reflection events within radargrams, but in correctly mapping these events to their physical sources in the subsurface. A persistent disconnect exists between detected hyperbolic signatures and actual buried infrastructure (a phenomenon termed event) utility mismatch [64,71]. This mismatch manifests in multiple forms: single utilities generating multiple reflection events, geometrically complex utilities producing fragmented signatures, and non-utility subsurface features mimicking utility responses. Critically, this ambiguity persists regardless of detection algorithm sophistication, as it originates from electromagnetic wave physics and subsurface complexity rather than computational limitations [72,73]. Understanding these mismatch mechanisms is essential for realistic assessment of GPR reliability in utility mapping workflows.

### 5.1. One Utility, Multiple GPR Events

A single buried utility can generate multiple distinct reflection events in GPR data through several physical mechanisms. Primary reflections from the pipe–soil interface typically produce the dominant hyperbolic signature, but secondary phenomena frequently introduce additional events that complicate interpretation. Multiples and re-reflections occur when electromagnetic energy bounces between the utility surface and overlying interfaces (pavement layers or soil stratigraphy), creating delayed hyperbolic patterns that appear deeper than the actual utility [97]. These spurious events may be misinterpreted as separate buried objects, particularly in layered urban subsurface environments where impedance contrasts exist at multiple depths.

**Figure 10:**
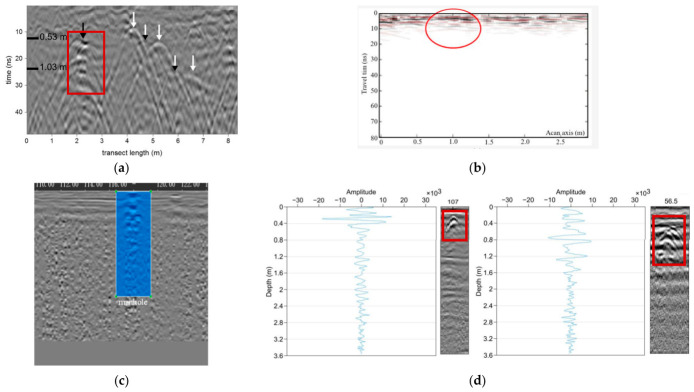
(**a**) Radar sections (the plastic pipes are shown in white with a black tip, the metal pipes in white, the pre-existing pipes in black) [97]; (**b**) radargram of mild steel water pipeline based on ageing 34 years [98]; (**c**) image of the radar data for measurement performed [99]; (**d**) the amplitude and hyperbolic shape of PVC pipe without leaks along some traces and the corresponding radar sections with leaks [100].

Composite responses further exacerbate ambiguity when utilities possess complex geometries or heterogeneous compositions (Figure 10). Corroded metallic pipes with irregular surface morphology scatter energy in multiple directions, producing distorted hyperbolas with amplitude modulations that deviate from idealized models [98]. Similarly, partially filled non-metallic pipes generate dual reflections, from both the pipe wall and the internal fluid interface, resulting in overlapping or nested hyperbolic patterns that resist straightforward parameter extraction [99]. Leakage plumes emanating from damaged water mains introduce additional complexity: moisture accumulation around pipe breaches alters local dielectric properties, creating diffuse reflection zones that extend laterally from the pipe axis and manifest as elongated or smeared hyperbolic signatures in B-scans [100,101,102].

### 5.2. Orientation Effects of Utilities Relative to Survey Direction

The geometric relationship between utility alignment and GPR survey trajectory critically determines reflection signature morphology, introducing substantial variability in detectability across adjacent survey lines. Utilities oriented perpendicular to the survey direction generate the characteristic symmetric hyperbolic response, as the antenna traverses the full spatial extent of the cylindrical target and captures the complete diffraction pattern [103]. This configuration maximizes reflection amplitude and produces hyperbolas with well-defined apex points that facilitate depth estimation.

Conversely, obliquely oriented utilities (typically defined as alignment angles between 15° and 75° relative to the survey line) yield asymmetric, truncated hyperbolas with reduced curvature and diminished amplitude [94,104]. The hyperbolic apex shifts laterally from the utility’s true subsurface projection, and the reflection energy distributes unevenly across the B-scan profile. When the utility runs nearly parallel to the survey line (alignment angle < 15°), the hyperbolic signature may fragment into a series of weak, quasi-horizontal linear reflections or disappear entirely beneath background noise levels [105]. Thakur and Prashant demonstrated through controlled experiments that hyperbola amplitude decreased by 40–60% when pipe orientation shifted from perpendicular (90°) to oblique (45°) trajectories, with detection probability dropping below 30% at angles less than 30° [56].

A utility appearing as a clear hyperbola in one profile might manifest as a faint linear feature or remain undetected in the neighboring profile merely 0.3 m away. Three-dimensional GPR arrays partially mitigate this issue by capturing spatial continuity across multiple orientations simultaneously, yet interpretation remains challenging when utilities exhibit complex routing with frequent directional changes [106,107]. Recent advances in orientation estimation using dual-polarized antennas show promise for quantifying alignment angles directly from reflection characteristics, though field validation under heterogeneous soil conditions remains limited [108].

### 5.3. Utility-like Events Without Utilities

**Figure 11:**
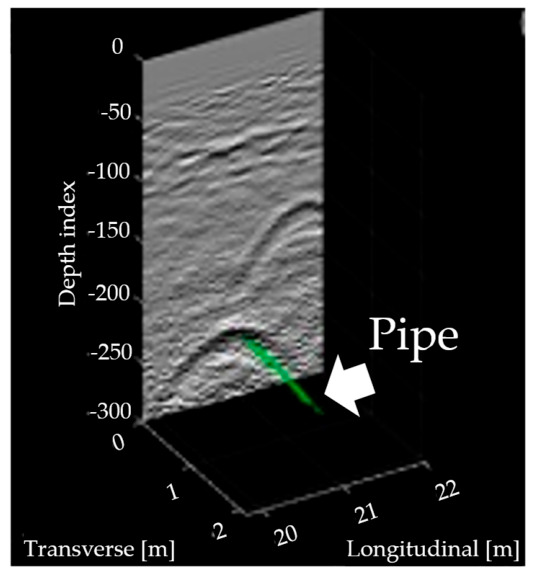
Pipes buried at a depth of 1 m from the ground surface [55].

Perhaps the most consequential source of event–utility mismatch arises from subsurface features that generate hyperbolic or quasi-hyperbolic signatures despite lacking any utility infrastructure (Figure 11). Natural geological heterogeneities frequently produce false-positive detections: isolated rocks or boulders within soil matrices create point-like diffractions indistinguishable from small-diameter pipes in single B-scans [55]. Voids, including animal burrows, root channels, or construction-induced air pockets, generate strong negative reflections that manifest as inverted hyperbolas easily confused with metallic utilities when polarity conventions vary across processing workflows [73]. Stratigraphic features such as lens-shaped clay pockets or abrupt soil texture transitions can produce curved reflections mimicking utility signatures, particularly when layer boundaries exhibit localized undulations [109].

**Figure 12:**
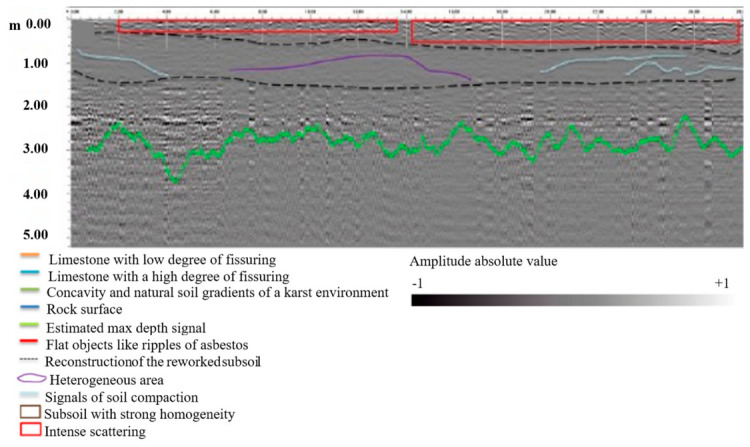
Images of some waste found during the excavation in the study site [110].

As Figure 12 illustrated, anthropogenic clutter further compounds ambiguity in urban environments. Construction debris (rebar fragments, brick rubble, asphalt chunks) scatters GPR energy into complex diffraction patterns that fragment into multiple hyperbolic segments across adjacent traces [110]. Excavation backfill zones with heterogeneous compaction create velocity variations that distort reflection geometries, generating pseudo-hyperbolic artifacts through wavefront focusing effects even in the absence of discrete targets [111]. System-induced artifacts including antenna ringing, cross-talk in multi-channel arrays, and migration smiles from incorrect velocity models introduce additional spurious events that automated detectors may classify as utilities [112]. Field studies comparing GPR detections with subsequent excavation revealed that 25–40% of algorithmically identified “pipes” corresponded to non-utility objects, with rocks and debris accounting for the majority of false positives in shallow urban surveys (<1.5 m depth) [91,92].

### 5.4. Implications for Interpretation and Automation

The pervasive nature of event–utility mismatch imposes fundamental constraints on both manual interpretation and automated detection systems. False positives (detecting utilities where none exist) lead to unnecessary avoidance zones during excavation planning, increasing project costs through rerouting of construction activities or excessive hand-digging verification [113]. More critically, missed utilities (false negatives) pose direct safety hazards when excavation equipment encounters undetected infrastructure, potentially causing service disruptions, environmental contamination, or catastrophic failures in pressurized systems [3]. The orientation-dependent detectability of utilities means that negative GPR results cannot reliably confirm absence of infrastructure, yet this limitation is frequently overlooked in field practice where “no detection” is misinterpreted as “no utility” [71].

Automation efforts face an inherent trade-off: algorithms tuned for high sensitivity to minimize missed utilities inevitably increase false positive rates by detecting non-utility hyperbolic features, while specificity-focused approaches reduce false alarms at the cost of missing obliquely oriented or deeply buried utilities [78]. Deep learning models trained predominantly on synthetic data, where utility signatures are idealized and clutter is minimized, exhibit particularly poor generalization to field conditions where mismatch phenomena dominate [88,89]. Recent domain adaptation techniques show modest improvements but cannot eliminate ambiguity arising from fundamental physical limitations such as the diffraction limit restricting resolution of closely spaced parallel utilities [11].

Consequently, responsible GPR application requires explicit acknowledgment of event–utility mismatch in decision-making frameworks. Best practices include: (1) integrating GPR with complementary locating technologies (electromagnetic locators, inertial mapping) to cross-validate detections [114]; (2) implementing confidence scoring that quantifies orientation uncertainty and clutter interference rather than binary detection outputs [115]; (3) mandating verification excavations in critical zones regardless of GPR results, recognizing that negative findings carry significant uncertainty [30]; and (4) documenting subsurface conditions that exacerbate mismatch (rocky soils, high-conductivity clays) to contextualize interpretation reliability [116]. Ultimately, computational advances can enhance event detection but cannot resolve the inverse problem’s inherent non-uniqueness, only multimodal data integration and cautious engineering judgment can mitigate the risks associated with event, utility mismatch in subsurface utility mapping.

Table 2 summarizes the main types of event–utility mismatch, their underlying physical causes, and their typical visual manifestations in B-scans.

## 6. Synthetic Data in GPR Utility Detection: Benefits and Domain Gaps

### 6.1. Drivers of Synthetic Data Adoption

**Figure 13:**
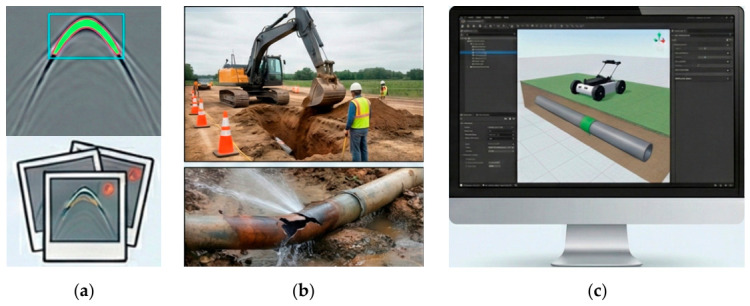
Drivers of synthetic data adoption. (**a**) Label scarcity; (**b**) cost and risk of excavation-based ground truth; (**c**) synthetic data facilitates controlled experimentation.

The adoption of synthetic data has become pervasive in GPR utility detection research, primarily driven by three interrelated challenges inherent to subsurface sensing applications (Figure 13). First, the scarcity of labeled datasets constitutes a fundamental bottleneck for training deep learning models. Manual annotation of GPR radargrams requires expert interpretation to distinguish hyperbolic signatures of utilities from clutter and noise, a labor-intensive process that severely limits dataset scale [117,118]. As Hu [117] notes, annotated datasets for deep learning applications in bridge deck inspection remain particularly limited despite the widespread deployment of GPR systems.

Second, establishing ground truth through excavation presents substantial practical constraints. Excavation is not only costly and time-consuming but also introduces safety risks and potential damage to existing infrastructure [116]. These constraints make comprehensive validation of detection algorithms across diverse subsurface conditions impractical. Consequently, researchers increasingly rely on synthetic data to circumvent the need for physical verification while still enabling algorithm development and preliminary validation [119].

Third, synthetic data facilitates controlled experimentation that would be impossible in field settings. Numerical simulations allow systematic variation in individual parameters, such as pipe diameter, burial depth, soil permittivity, or antenna frequency, while holding all other factors constant [120]. This capability proves invaluable for sensitivity analyses, algorithm benchmarking under known conditions, and understanding the physical relationships between subsurface properties and radar responses [13]. For instance, Wu and Sheil [121] leveraged controlled synthetic environments to develop a stochastic-ellipse-union method for modeling robot-constructed underground structures with mathematical precision before field validation.

### 6.2. Common Synthetic Data Generation Practices

Contemporary synthetic GPR data generation predominantly relies on electromagnetic simulation techniques, with the finite-difference time-domain (FDTD) method implemented in tools like gprMax serving as a common foundation [118,122]. Warren et al. further noted that half-space dipole configurations are a canonical starting point for many GPR simulations because near-field interactions among the antenna, the ground, and subsurface targets play a central role in shaping the response [123]. These simulators solve Maxwell’s equations numerically to model wave propagation through user-defined subsurface geometries, producing B-scan images that replicate the hyperbolic diffraction patterns characteristic of cylindrical utilities [124]. The workflow typically involves constructing a 2D or 3D model specifying dielectric properties of soil layers and buried targets, then simulating antenna movement across the surface to generate sequential A-scans that form the radargram [125].

Despite their physical basis, most synthetic generation pipelines incorporate significant simplifications to balance computational feasibility with realism. Soil models frequently assume homogeneous or horizontally stratified media with constant permittivity values, neglecting the spatial heterogeneity characteristic of natural subsurface environments [126]. Target representations similarly prioritize geometric simplicity, typically modeling pipes as perfect cylinders with uniform electromagnetic properties, while omitting complexities like corrosion, sediment accumulation, or partial infilling that alter radar signatures in practice [127]. Antenna responses are often idealized through analytical approximations rather than full-wave electromagnetic modeling of actual antenna structures, potentially misrepresenting near-field effects and radiation patterns [89].

Recent advances have sought to bridge these simplifications through hybrid approaches. Guo et al. [15] proposed a two-step forward modeling strategy combining image translation (via a Polarization Self-Attention Image Translation network) with style transfer to convert clutter-free simulated images into data matching real-world heterogeneous medium characteristics. Similarly, Xu et al. [127] developed FM-GAN, a forward modeling generative adversarial network incorporating transformer fine-tuning to enhance adaptability to real environments. Chen et al. [89] demonstrated denoising diffusion probabilistic models (DDPMs) for generating high-resolution GPR images with substantial diversity and feature realism. Nevertheless, these methods still depend fundamentally on simplified initial models before applying domain adaptation techniques.

### 6.3. Sources of the Synthetic–Field Domain Gap

A critical limitation of conventional synthetic data lies in the persistent domain gap between simulated and field-collected GPR measurements—a disparity that undermines model transferability. Yao et al. [118] explicitly acknowledged the substantial disparity between directly simulated gprMax data and actual GPR images, necessitating novel synthesis methods to bridge this gap. Three primary factors contribute to this disconnect:

First, soil heterogeneity and moisture dynamics remain inadequately represented in simulations. Real subsurface environments exhibit complex spatial variations in dielectric properties due to soil composition gradients, moisture content fluctuations, and organic matter distribution—factors that significantly influence wave velocity and attenuation [119]. While FDTD methods can theoretically model heterogeneous media, obtaining precise spatial distributions of permittivity and conductivity for realistic simulation remains impractical [15]. As Guo et al. [15] observed, traditional numerical methods face challenges in accurately simulating complex heterogeneous mediums in real-world scenarios due to the difficulty of obtaining precise medium distribution information. Third, operator- and system-dependent artifacts introduce variability unaccounted for in idealized simulations. These include variations in antenna coupling due to surface roughness, inconsistent survey speeds affecting spatial sampling density, cable sway during data collection, and system-specific noise characteristics [22]. Patsia et al. [22] demonstrated that cross-coupling between transmitter and receiver antennas creates background responses that mask weak target signals effects difficult to model without precise antenna characterization. Furthermore, environmental factors like temperature variations affecting electronics performance or precipitation altering near-surface dielectric properties introduce temporal dynamics absent in static simulations [128].

### 6.4. Case Study

**Figure 14:**
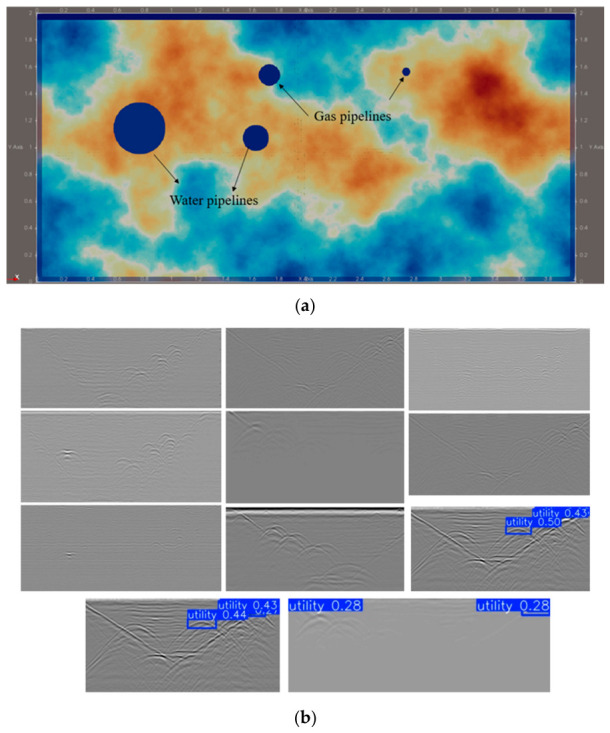
(**a**) Underground pipeline GPR B-scan diagram; (**b**) Representative testing result on real-world GPR radargrams, showing the limited transferability of a model trained on simulated data.

To model subsurface utility detection scenarios, this study simulated gas pipelines (plastic construction, dielectric constant *ε_r_* = 3) and water mains (metallic construction, *ε_r_* → *∞*). Gas pipelines were positioned at burial depths of 0.30–0.38 m with diameters of 0.05–0.20 m, while water mains occupied depths of 0.60–1.50 m with diameters of 0.15–0.40 m. Each simulation stochastically generated 2–5 pipes within these parameter bounds, with randomized burial depths and diameters drawn from uniform distributions. The computational domain spanned 4.0 m (*x*) × 2.0 m (*z*), discretized using a 4 mm isotropic grid (Δ*x* = Δ*y* = Δ*z* = 0.004 m). Soil heterogeneity was incorporated through spatially varying dielectric properties to enhance field fidelity. Simulations employed a 60 ns time window with a 0.55 m antenna offset. A zero-phase Ricker wavelet (peak amplitude = 1.0, central frequency = 400 MHz) served as the source signal. Antenna movement followed a 8 mm step increment, with 475 consecutive A-scans coherently stacked to generate the final B-scan profile (Figure 14a).

To validate the effectiveness of the proposed predictive model, this study utilizes the publicly available GPR dataset established by Dérobert and Pajewski [129] for testing. For this study, 11 radar images exhibiting typical hyperbolic reflection signatures were selected from the dataset to evaluate the accuracy of the proposed prediction model in identifying different subsurface targets. Based on the YOLO11m model, automatic detection of underground pipelines was implemented. Further testing of the trained model reveals that it could only correctly identify targets in 3 out of the 11 images and failed to detect all hyperbolic signatures present in the images (Figure 14b). Consequently, the automated identification and prediction model trained on simulated data exhibits limited capability in interpreting GPR images acquired from real-world environments. Although heterogeneous soil conditions were simulated during the modeling process, the simulation could not fully replicate the actual noise interference encountered in field conditions. This discrepancy leads to errors during the model’s recognition process and, in some cases, results in a complete failure to detect targets.

### 6.5. Consequences for Learning-Based Models

The synthetic–field domain gap manifests in three detrimental consequences for learning-based utility detection systems. Most critically, models trained exclusively on idealized synthetic data tend to overfit to simplified signature representations, failing to generalize when confronted with the nuanced variations present in field data. Giannakis et al. [130] demonstrated that hyperbola fitting (technique effective in synthetic environments) becomes unreliable for simultaneously estimating both medium velocity and target size in practical surveys due to inherent non-uniqueness and noise sensitivity. Similarly, conventional approaches assuming perpendicular antenna traverses relative to pipe orientation yield significant errors when this assumption is violated in field conditions, necessitating angle-corrected models as proposed by He and Lai [71].

Second, cross-site and cross-system generalization remains problematic. Models achieving high accuracy on synthetic data or site-specific field data often degrade substantially when deployed in new locations with different soil properties or using different GPR hardware [131]. Jafary et al. [116] explicitly designed their 3D reconstruction pipeline to avoid synthetic augmentation precisely because prior studies suffer from poor generalizability to complex real-world scenarios and reliance on full 3D data volumes. Transfer learning strategies have shown promise in mitigating this issue; Hu [117] demonstrated that pretraining YOLO models on simulated GPR data before fine-tuning on limited real data improved detection accuracy compared to conventional COCO pretraining, validating domain-specific transfer learning as a partial remedy.

Third, performance metrics derived solely from synthetic data can be misleadingly optimistic. Han et al. [122] observed that while forward modeling simulation enhanced training precision and recall, the proportion of data augmentation should not be too high indicating diminishing returns and potential negative transfer when synthetic data dominates training sets. Their experiments further revealed that CycleGAN-based transformation between measured and simulated images significantly improved recognition accuracy, suggesting that unmodified synthetic data alone provides insufficient training signal. These findings align with Zhu et al. [120], who incorporated domain randomization in their TunGPR framework specifically to enhance robustness against dielectric property variations encountered in real tunnel environments.

In conclusion, these limitations underscore the necessity of hybrid data strategies that thoughtfully integrate synthetic and real measurements. Recent successful approaches—including Yao et al.’s [118] synthesis of gprMax-generated data with real measurements, Wu and Sheil’s [121] combination of calibrated synthetic data with real-world pipe reflections, and Guo et al.’s [15] style transfer technique—demonstrate that bridging the domain gap requires explicit modeling of the transformation between idealized simulations and field conditions rather than treating synthetic data as a direct substitute for real measurements.

## 7. Toward Reliable Utility-Level Inference

The transition from detecting isolated subsurface anomalies to inferring continuous utility networks represents a critical evolution in GPR interpretation. While contemporary deep learning models excel at identifying hyperbolic signatures, reliable infrastructure management requires reasoning about the utility as a coherent physical entity. This chapter outlines a framework for achieving utility-level inference, emphasizing spatial continuity, physical consistency, domain adaptability, and uncertainty quantification.

### 7.1. From Event Detection to Utility Reasoning

Traditional GPR analysis often treats subsurface targets as discrete events, neglecting the spatial continuity inherent in linear infrastructure such as pipelines and cables. To achieve reliable utility-level inference, detection frameworks must evolve to incorporate multi-event aggregation and spatial consistency constraints. Recent advancements demonstrate that integrating multi-view features significantly enhances localization accuracy. For instance, frameworks utilizing B/C/D-scan three-view joint analysis strategies have established 3-D pipeline feature evaluation methods that resolve inherent ambiguities in single-view detection [132]. By cross-validating forward simulation results with actual measurement data, these systems achieve robust 3-D localization through lightweight joint analysis of multi-view 2-D GPR images.

Beyond multi-view fusion, reconstructing the spatial trajectory of buried utilities requires clustering detected features into continuous paths. Approaches employing 3D DBSCAN algorithms to cluster detected summit points, followed by RANSAC-based line fitting, have shown efficacy in approximating spatial trajectories with low root-mean-square error [116]. Automated systems further refine this by estimating key parameters such as two-way travel time, burial depth, and pipeline diameter directly from hyperbolic reflections, reducing reliance on manual inspection [133]. However, single-sensor data often lacks the contextual information necessary for comprehensive mapping. Consequently, probabilistic pipeline mapping models that fuse multisource data, including statutory records, manhole covers, and remote sensing technologies, have been proposed to iteratively classify and fit detected points into coherent pipeline maps [134].

**Figure 15:**
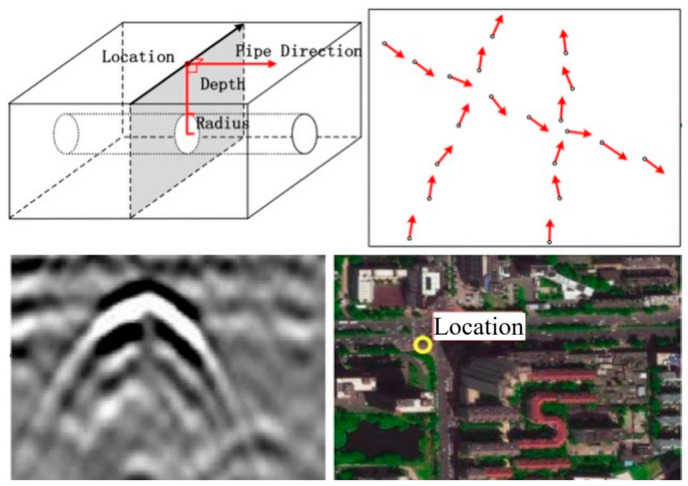
Example of a probabilistic utility-level mapping framework that integrates GPS and GPR data to infer pipe locations and directions from detected subsurface responses [139].

The integration of heterogeneous data sources is paramount for verifying utility existence and configuration. Robotic subsurface pipeline mapping methods that fuse GPR scans with camera images and visual simultaneous localization and mapping (V-SLAM) outputs enable the simultaneous detection of multiple pipelines without requiring perpendicular scanning trajectories [7]. The transition from event detection to utility-level reasoning also depends on accurate spatial registration of radar observations. Ferrara et al. demonstrated that integrating GPR with GPS and IMU improves buried-object localization by compensating for route and attitude variations, highlighting that reliable mapping requires not only signal interpretation but also sensor-level control of positioning geometry [135]. Furthermore, mapping buried cables using GPR combined with Gaussian-process regression allows for the derivation of confidence intervals for cable locations, accounting for position and depth noises [136]. To address the issue of unreliable legacy data, integration-driven data reconciliation approaches argue for combining as-built GIS records, GPR scans, and open excavation 3D scans to verify and update subsurface utility data throughout the infrastructure life cycle [137]. Information fusion approaches based on Dempster-Shafer evidence theory further enhance confidence by integrating sensing and non-sensing data, significantly reducing location estimation errors compared to single-source methods [138]. Probabilistic mixture models have also been developed to map buried pipes by classifying detecting points into different classes and connecting them to accurate pipeline locations, ensuring convergence even in intersected pipe areas (Figure 15) [139].

### 7.2. Physics-Guided and Hybrid Approaches

Purely data-driven models often struggle with generalization when faced with subsurface conditions divergent from training distributions. Embedding electromagnetic (EM) priors and physical constraints into learning architectures offers a pathway to improve physical plausibility and robustness. Physics-guided conditional diffusion models have been proposed to integrate physical prior constraints with deep learning, ensuring that denoised GPR results conform to electromagnetic propagation principles via wave equation constraints [140]. This integration significantly improves noise suppression and signal reconstruction in complex environments.

Hybrid approaches combining traditional inversion algorithms with deep learning leverage the strengths of both methodologies. A synergistic GPR approach integrating the back projection (BP) algorithm and FWI optimizes reconstruction outcomes by using BP estimation results as a reliable initial model for FWI, thereby reducing the risk of local minima [141]. Similarly, deep learning inversion models based on embedded velocity fields utilize estimated global velocity information within the detection domain to reconstruct subsurface permittivity distribution, demonstrating superior performance in complex underground scenes compared to models lacking prior velocity information [142]. The application of Physics-Informed Neural Networks (PINNs) to model GPR observations on railway tracks suggests that exploiting Maxwell’s equations can approximate underlying electromagnetic wave field propagation, although challenges remain in layered media implementations [143].

Advanced inversion frameworks continue to evolve to handle specific utility defects. Dual-input image-wise separable Attention U-Net models designed for GPR data inversion focus on detecting small-sized objects and identifying material composition by integrating B-scan and F-K images [144]. For leakage characterization, attention-guided GPR inversion frameworks enable refined imaging of leakage plumes and their temporal-spatial evolution, leveraging dual advantages of Convolutional Block Attention Modules and U-Net architectures [145]. Additionally, Bayesian waveform inversion frameworks using finite-difference time-domain simulators and discrete cosine transforms have demonstrated success in detecting defects in underground concrete structures, showing insensitivity to noise levels as long as sufficient measurements are available [146].

### 7.3. Domain-Aware Learning Strategies

The deployment of GPR interpretation models across diverse geological and environmental conditions necessitates domain-aware learning strategies. Transfer learning and domain adaptation are critical for mitigating the scarcity of annotated field data. Model-based transfer learning approaches for 3D GPR cavity identification utilize pre-training on simulated data followed by fine-tuning with a small amount of real underground cavity data, breaking through limitations of insufficient sample sizes [147]. In scenarios involving varying soil properties, multi-model knowledge transfer frameworks detect targets in different media by training classifiers on ample data from known soil types and adapting them to unknown sources with scarce training data [148].

Multitask learning further enhances adaptability by stitching soil and target classification together, allowing customized classifiers to detect targets according to soil type, which yields superior classification rates compared to standard convolutional neural networks [149]. Pre-training CNNs on large datasets of grayscale imagery, such as aerial imagery or CIFAR-10, and using subsets of parameters to initialize buried threat detection networks has been shown to improve performance where labeled GPR examples are few [150].

To address data scarcity, generative models are increasingly employed for field-informed augmentation. Combined image-guided generative adversarial networks (CIGGAN) generate GPR data with voids by combining various voids and backgrounds, enhancing feature diversity and improving detection F1 scores when added to original datasets [151]. Dense Generative Adversarial Networks have also been proposed to simulate GPR B-scan images with high similarity to real data, contributing to the intelligent processing of GPR data where labeled images are shortage [87]. Unsupervised domain adaptation methods, such as improved Cycle-Consistency Generative Adversarial Networks, convert clutter images into clutter-free images via style transfer, significantly improving improvement factors in measured data [152]. Furthermore, unsupervised deep learning image-to-image translation methods tailored for GPR images allow models trained on simulated data to directly identify defects in real GPR images without labeling real data, preventing semantic distortion through geometry-consistency constraints [153].

Despite their strong representational capacity, many advanced utility-level inference frameworks remain difficult to deploy directly in routine field operations. Models such as PINNs, attention-enhanced U-Net variants, diffusion-based networks, and Transformer-based architectures often require substantial computational resources for training and, in some cases, also for inference, particularly when processing high-density multi-view or 3D GPR data. In practice, mobile GPR carts and vehicle-mounted systems typically operate under constraints in onboard computing power, memory, energy supply, heat dissipation, and real-time data throughput. These limitations have motivated recent efforts toward lightweight model design and complexity reduction for GPR applications [16]. At the same time, advanced inversion models based on attention-guided U-Net frameworks still involve non-negligible processing costs, which may restrict their direct use in edge environments requiring rapid interpretation [144]. As a result, there remains a practical gap between algorithmic sophistication and field deployability. Future progress should therefore consider not only inversion accuracy and physical consistency, but also model efficiency, deployability, and the feasibility of real-time implementation in operational surveying workflows.

### 7.4. Uncertainty-Aware Utility Mapping

Reliable utility inference requires not only accurate predictions but also quantified uncertainty to support risk-informed decision-making. Probabilistic frameworks provide the necessary confidence estimates for engineering applications. Machine learning models integrated with Monte Carlo simulations quantify uncertainties associated with input parameters, visually representing results through load–settlement curves with 95% confidence intervals [154]. Bayesian optimization frameworks address gaps in back-analyzing numerical model parameters from radar data, using Gaussian Process Regression as a surrogate model to minimize simulations while determining best-fitting coefficients [155].

From a civil engineering perspective, the value of uncertainty quantification lies in how it informs field action rather than in the statistical output itself. For example, a predicted utility position accompanied by a narrow confidence interval may justify routine excavation with standard clearance procedures, whereas a wider interval should trigger more conservative responses, such as enlarging the safety buffer, reducing excavation speed, restricting the use of heavy equipment, or requiring verification by potholing or complementary sensing before intrusive work begins [154]. In this sense, probabilistic outputs can be translated into tiered decision rules: low-uncertainty detections support direct planning, moderate-uncertainty detections call for caution and additional confirmation, and high-uncertainty detections should be treated as risk zones until resolved. This is particularly important in dense urban settings, where even limited localization error may lead to utility strikes, service interruption, or unnecessary avoidance measures [156]. Therefore, future uncertainty-aware utility mapping frameworks should not only report confidence intervals or posterior distributions, but also link them to explicit operational thresholds for clearance, verification, and excavation control.

Probabilistic inverse analysis methods integrating Bayesian inference and Bidirectional Long Short-Term Memory models efficiently estimate dielectric constants and thicknesses of multi-layer soils while effectively quantifying associated uncertainties [157]. In the context of threat detection, probabilistic models based on queuing theory analyze and design detection systems operating with multistate management, accurately predicting system behavior and validating utility for designing multimodality systems [158]. Bayesian inversion methods for cross-hole GPR data reconstruct relative permittivity fields, where the posterior uncertainty of reconstructed fields is managed by adjusting the number of coefficients used to characterize the structure [146].

Evidence reasoning rules incorporating evidence reliability as a random variable following a Gaussian distribution construct joint reasoning models for obtaining reasonable evaluation results, enhancing effectiveness in handling uncertainties compared to quantitative value treatments [159]. Finally, accurate mapping of underground utilities using information fusion approaches assesses the trust level of fused results, proving that non-sensing data provides valuable information for spatial reasoning and should not be ignored in uncertainty management [160]. By embedding these probabilistic measures into utility mapping workflows, stakeholders can transition from deterministic detections to risk-aware infrastructure management.

## 8. Conclusions

The comprehensive analysis presented in this review underscores the pivotal role of GPR in modern subsurface infrastructure management, while highlighting the persistent barriers preventing its transition from a diagnostic tool to an autonomous mapping system. Bibliometric trends confirm a rapid acceleration in algorithmic research, particularly within deep learning architectures, yet this scholarly output contrasts with the slower pace of practical field adoption. The technical synthesis indicates that while computational methods have significantly enhanced the visibility of reflection events, the core reliability of utility detection remains bounded by physical ambiguities and data fidelity issues rather than processing capabilities alone.

Central to these limitations are two fundamental gaps identified throughout the literature. The first is the event–utility mismatch, where current detection frameworks prioritize the identification of hyperbolic signatures over the inference of continuous utility networks. As demonstrated in the analysis of field data, electromagnetic responses are non-unique; a single pipe may generate multiple reflection events due to structural complexities or leakage, while natural clutter often produces false positives indistinguishable from utilities in single-view B-scans. Consequently, high accuracy in event detection does not equate to reliable infrastructure mapping. The second critical barrier is the synthetic–field domain gap. The widespread dependence on numerical simulations, such as those generated by gprMax, facilitates algorithm training but fails to capture the stochastic heterogeneity of urban soils and system-specific noise. Models optimized on idealized synthetic data frequently suffer performance degradation when deployed in real-world surveys, creating a disparity between laboratory metrics and operational effectiveness.

Resolving these issues necessitates a strategic shift in evaluation protocols, data generation practices, and interpretation goals. Future developments should prioritize utility-level reasoning that enforces spatial continuity and physical consistency, moving beyond isolated event classification. Evaluation standards must evolve to include field validation metrics that quantify uncertainty and orientation sensitivity, rather than relying solely on synthetic benchmark accuracy. Data practices should transition toward hybrid strategies that combine physics-informed generative models with curated, open-access field datasets to bridge the simulation-to-reality divide. Ultimately, advancing GPR technology requires acknowledging the inherent non-uniqueness of electromagnetic inverse problems. By aligning algorithmic development with physical realism and focusing on utility-level inference, the research community can deliver systems that provide not merely detections, but actionable, risk-aware intelligence for subsurface engineering.

## Figures and Tables

**Table 1:** Comparison of different proposed models’ performance.

Study	Application	Dataset	Image Size	Algorithm	Performance
Ishitsuka et al. [61]	Road	305	65 × 65 pixels	Deep CNN	Accuracy: 0.945 Ture Positive Ratio (TPR): 0.741 Ture Negative Ratio (TNR): 0.964
Liu et al. [81]	PVC Pipeline	1000	512 × 512 pixels	Attention-Driven Deep Networks	IoU = 0.668 Root-Mean-Square Error (RMSE): 0.644 Mean Absolute Error (MAE): 1.071 Multiscale Structural Similarity (MS-SSIM): 0.983
Panda et al. [82]	Metal Cylinders	6000	256 × 64 pixels	Attention U-Net	Improvement Factor (IF): 34.7514 dB Signal-to-Noise Ratio (SNR): 35.5678 Structural Similarity (SSIM): 0.9945
Jin et al. [83]	Seabed Pipelines (Seabed GPR Dataset)	124	1280 × 720 pixels	Transformer-based neural network	True hyperbola-point precision (TP_Pre): 0.867 True hyperbola-point recall (TP_Rec): 0.762 TP_F1: 0.811
	Building Areas (CMU-GPR)	283			TP_Pre: 0.402 TP_Rec: 0.736 TP_F1: 0.520
	Underground Pipelines (GPR-Classification)	1495			TP_Pre: 0.744 TP_Rec: 0.723 TP_F1: 0.733
Li et al. [17]	Cavity, Void, Loose, Predicted Pipelines, Manhole Covers, and Metal Net	1044	N/A	YOLOv11-CAFM	Precision: 0.840 Recall: 0.850 mAP50: 0.881 mAP50:95: 0.584
Li et al. [84]	Pipes	7467	448 × 448 pixels	MV-GPRNet	Precision: 1.00 Recall: 1.00 F1: 1.00
Wang et al. [85]	Underground Pipeline	4330	N/A	CycleGAN	MSE: 0.006 PSNR: 34.73 dB
Hou et al. [86]	Pipes, Voids, Cavities	1617	512 × 512 pixels 128 × 128 pixels	Dual Attentive YOLOv11 Keypoint Detector	Precision: 0.937 mAP50: 0.947 mAP50:95: 0.825 F1: 0.929

**Table 2:** Summary of major event–utility mismatch types in real-world GPR data.

Mismatch Category	Typical Physical Cause	Visual Manifestation in B-Scans	Practical Interpretation Risk
One utility producing multiple events [97]	Multiples and re-reflections between the utility and overlying interfaces such as pavement layers or stratified soils.	Additional delayed hyperbolic responses appearing deeper than the true utility.	Secondary events may be mistaken for separate buried objects.
One utility producing composite or distorted signatures [98,99]	Irregular geometry, corrosion, heterogeneous materials, or partially filled pipes with multiple internal interfaces.	Distorted, overlapping, nested, or amplitude-modulated hyperbolas.	Utility depth, size, or number may be misinterpreted.
Utility with leakage-related response extension [100,101,102]	Moisture accumulation around damaged pipes changes local dielectric properties.	Smeared, broadened, or laterally extended hyperbolic zones.	Diffuse anomalies may be wrongly interpreted as multiple targets or clutter.
Orientation-induced mismatch [56,94,104,105]	Utility alignment is oblique or nearly parallel to the survey direction.	Asymmetric, truncated, weakened hyperbolas; quasi-horizontal reflections; in some cases no clear hyperbola.	A real utility may be missed or incorrectly localized.
Cross-profile variability of the same utility [106,107]	Small changes in survey line position relative to the utility axis and orientation.	Clear hyperbola in one profile but faint linear or fragmented response in adjacent profiles.	Inconsistent detections across profiles may reduce confidence or lead to false negatives.
Non-utility object mimicking a utility [55,73,109]	Rocks, boulders, voids, root channels, or localized geological heterogeneity.	Hyperbolic or quasi-hyperbolic signatures similar to pipe responses.	False positives, especially in cluttered shallow subsurface conditions.
Anthropogenic clutter resembling utilities [91,92,110,111]	Construction debris, rebar fragments, rubble, and disturbed backfill.	Fragmented diffraction patterns or pseudo-hyperbolic reflections.	Clutter may be classified as utilities by manual or automated interpretation.
System- or processing-induced artifacts [112]	Antenna ringing, cross-talk, or migration artifacts caused by incorrect velocity assumptions.	Spurious curved events or artificial hyperbola-like features.	Artifacts may be falsely interpreted as buried infrastructure.

## Data Availability

The original contributions presented in this study are included in the article. Further inquiries can be directed to the corresponding author.
